# Models for correction of myopia in the South Asia region

**Published:** 2019-05-13

**Authors:** GVS Murthy

**Affiliations:** 1 Director: Indian Institute of Public Health, Hyderabad, India & Professor, Public Health Eye Care & Disability, LSHTM, London, UK.

**Models for correction of myopia need to target identification and correction of those with myopia on the one hand and interventions for modifiable factors to prevent onset and slow down progression on the other.**

The prevalence of myopia is increasing in South Asia with earlier onset and high progression rates. Among school-aged children, population studies indicate a prevalence of myopia of (<= −0.5 D) ranging from 1.2–7.4 per cent while school-based studies indicate a higher prevalence between 6–10 per cent in the South Asia region.^1^ Among adults, prevalence rates have varied from 17 per cent to 42.7 per cent (<-0.5 D).^2^

Models for correction of myopia need to target identification and correction of those with myopia on the one hand and interventions for modifiable factors to prevent onset and slow down progression on the other. The modifiable factors are mostly related to nurture. Controlling the modifiable factors is also dependent on whether it is at a clinic level or at a programme level. Some interventions such as use of pharmacologic agents like low-dose atropine are more suited for clinic-based interventions (e.g. percentage (%) atropine drops instilled in the eye every alternate day), while others such as encouraging children to spend more time outdoors (e.g. spending the recess time outside the class room for up to 11 hours a week) are a significant public health approach.

In the South Asia region where prevalence among children is low, it is operationally pragmatic to integrate such activities into overall physical health improvement to reduce the risk of obesity, non-communicable diseases like diabetes, along with reducing the risk of myopia. Such optimisation has comprehensive benefits which also impact vision and can be offered at a more affordable cost. Cost effectiveness of interventions needs to be carefully considered, especially when prevalence is low. In such a context, searching for myopia alone increases the cost of care compared to integrating vision screening in school health screening programmes.

South Asia has been the cradle of innovation in eye care. School vision screening programmes have been in vogue, especially in India, for more than three decades while comprehensive school health screening, including vision and hearing, has been established for more than 60 years.

## Programme models for myopia

Models for myopia correction can be categorised by population age

- 5–17 years
- 18–39 years
- 40+ years

Some models cater to all ages at the same time as is done in a population-screening camp model; this approach is opportunistic. Those aged 18–39 years, when symptomatic may attend an eye care facility that includes an optician for their vision correction. It is probably not efficient to organise an activity exclusively for this group as the numbers will be low unless it is done as part of occupational health in factories and work places.

**Figure:**
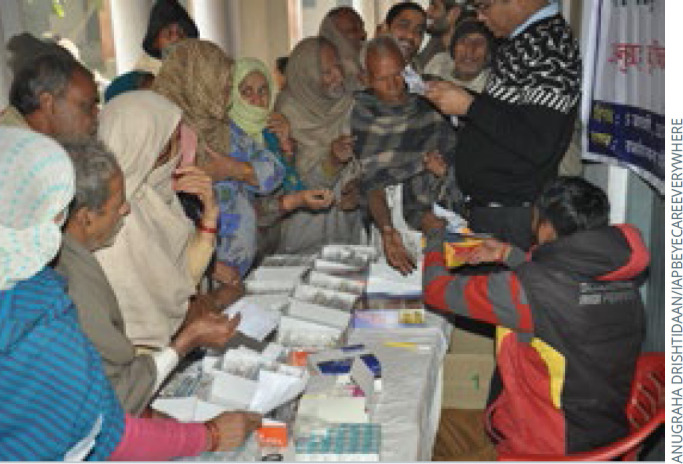
Villagers collecting their spectacles after eye check-up. INDIA

## Models for older populations

Evidence shows that 70 per cent of older age adults with myopia have cataract^3^; therefore the focus of any programmatic intervention for myopia should be a comprehensive eye service so that the underlying pathology can be managed effectively. Modalities like vision centres or comprehensive eye screening camps are appropriate options for this age group. Such an approach also ensures that people who are prescribed and dispensed spectacles do not go away with the notion that this ‘rectifies’ their underlying pathology. Additional challenges in this group is the loss to follow up and compliance with treatment advice.

## Models targeting children

The significant population segment that can benefit from a programmatic approach to myopia are school-aged children. In most countries of South Asia, school enrollment rates have increased significantly over the past two decades, except in difficult geographical terrain. When more than 80–90 per cent of children are in schools, it is logistically pragmatic and efficient to search for children with myopia in schools. Studies have shown that the prevalence of myopia is insignificant below the age of five years and increases between 11 to 15 years. This epidemiologic characteristic has led to the rationale of targeting children in grades from five to ten for school vision screening programmes.

### Identifying children with myopia

School eye screening programmes prioritise myopia detection as it is the most prevalent refractive error in this age group. Most of the studies both globally and in the South Asia region observed that the prevalence of childhood myopia is significantly higher in urban environments.^3^,^4^ This could be related to lifestyle factors and early exposure to near-work and parental pressure on academic achievements. Therefore the first priority in South Asia is children aged 11+ in urban schools as they have a higher risk of myopia.

Integrating vision screening in a school health programme is a cost-effective and sustainable approach compared to a stand-alone vision screening. In India the commonest approach has been training school teachers to do the initial vision test, followed by referral of those with ‘suspected abnormal vision’ to an ophthalmic assistant or optometrist. In some areas, all the school teachers have been trained so that the class teacher can undertake the screening. This is thought to improve compliance as the students are more comfortable with their regular teacher. Another approach involves trained ophthalmic personnel screening the children at school. This is not an affordable approach as there is a paucity of skilled eye care personnel in most of South Asia. Models have also been developed to reach school dropouts and out-of-school children in India.

In all these models, the essential parameter for success is provision of spectacles. If this is not part of a vision ‘screening’ initiative the entire exercise is futile. The modality of providing spectacles differs from one initiative to another. Most commonly, children are prescribed correction and this is provided through a designated optician. Some organisations dispense spectacles on the spot. Since wearing spectacles is a stigma in South Asian cultures, measures to improve compliance include provision of an array of colorful frames, and more recently, the use of smartphone-based screening (PEEK school eye health) where screened children, their guardians and teachers are shown a simulated sight on the smart-phone.^5^

**Figure:**
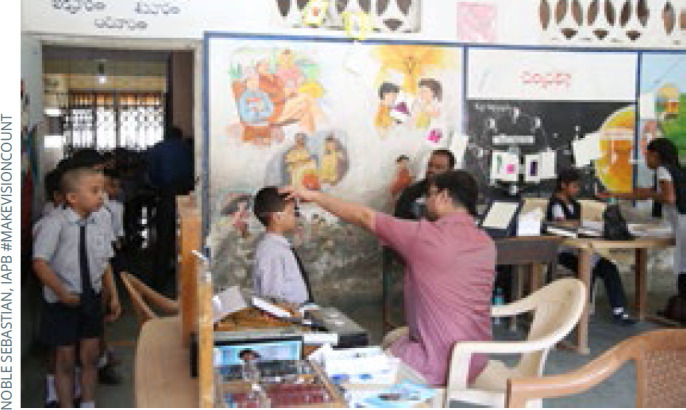
Children being screened at school. INDIA

### Prevention of myopia

Since environmental risk factors have been postulated to be responsible for significant increases in myopia there have been attempts to ‘modify’ this risk. Time spent outdoors and away from near work activities in a class room has been found to reduce the risk of myopia development and progression of myopia. Large scale trials of prevention have not been done in South Asia but outdoor activity is a promising intervention as it has a positive effect not only on myopia but also on reducing obesity and the risk of non-communicable diseases in later life. The use of pharmacologic agents like low-dose atropine are not warranted as a public health measure in South Asia where the prevalence of childhood myopia is low. It may however find use in a clinical setting when parents are willing to accept the option.

**Figure:**
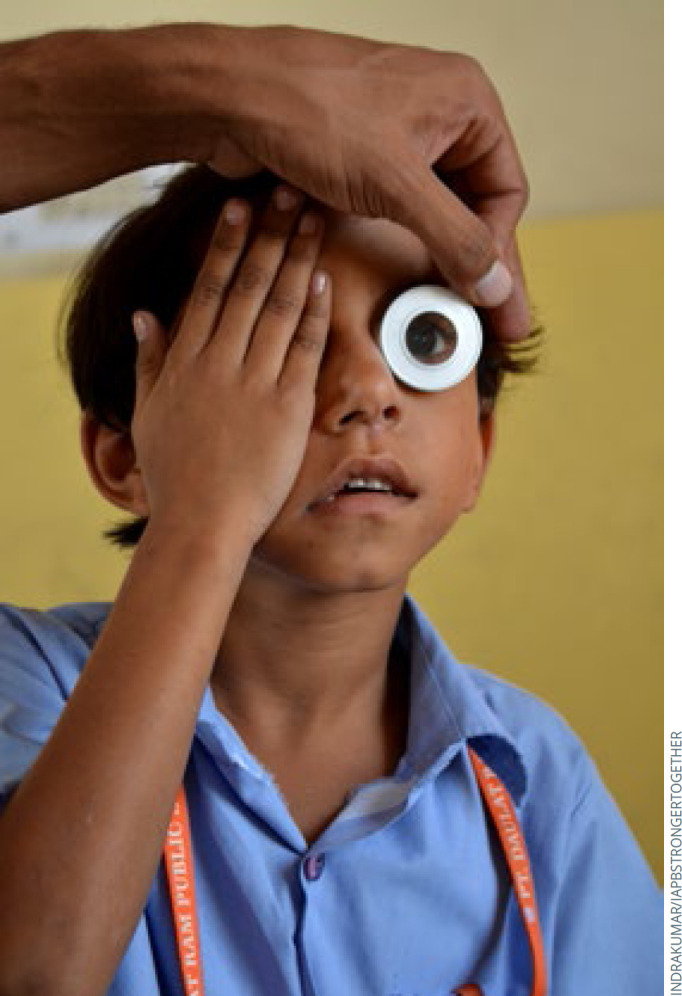
Identifying children with myopia. INDIA

## References

[1] Joseph S Krishnan T Ravindran RD et al. Prevalence and risk factors for myopia and other refractive errors in an adult population in southern India. Ophthalmic Physiol Opt 2018; 38:346–358 29574882 10.1111/opo.12447 PMC6001660

[2] Murthy GVS Gupta SK Ellwein LB Munoz SR et al. Refractive error in children in an urban population in New Delhi. Invest Ophthalmol Vis Sci 2002; 43: 623–631 11867576

[3] Rono HK Bastawrous A Macleoad D et al. Smartphone-based screening for visual impairment in Kenyan school children: a cluster randomized controlled trial. Lancet Global Health 2018; 6: 924–32 10.1016/S2214-109X(18)30244-4 PMC6057135 30012273

[4] Dandona R Dandona L Srinivas M Sabare P et al. Refractive error in children in a rural population in India. Invest Ophthalmol Vis Sci 2002; 43: 615–622 11867575

